# Supplementary material for: Structural Diversity and Carbon Dioxide Sorption Selectivity of Zinc(II) Metal-Organic Frameworks Based on Bis(1,2,4-triazol-1-yl)methane and Terephthalic Acid
Source: Molecules. 2022 Oct 1;27(19):6481. doi: 10.3390/molecules27196481 (PMC9571910; doi:10.3390/molecules27196481)

checkCIF/PLATON report

Structure factors have been supplied for datablock(s) tgs125

THIS REPORT IS FOR GUIDANCE ONLY. IF USED AS PART OF A REVIEW PROCEDURE FOR PUBLICATION, IT SHOULD NOT REPLACE THE EXPERTISE OF AN EXPERIENCED CRYSTALLOGRAPHIC REFEREE.

No syntax errors found. CIF dictionary Interpreting this report

Datablock: tgs125

Bond precision:	C-C = 0.0075 A		Wavelength=0.71073
Cell:	a=9.7232(13)	b=18.204(3)	c=18.626(3)
	alpha=117.577(4)	beta=102.500(5)	gamma=92.264(5)
Temperature:	150 K		

	Calculated	Reported
Volume	2815.6(8)	2815.6(7)
Space group	P -1	P -1
Hall group	-P 1	-P 1
Moiety formula	2(C32 H25 N7 O13 Zn3), 8(C1.50 H3.50 N0.50 O0.50) [+ solvent]	C32 H25 N7 O13 Zn3, 3.5(C3 H7 N O), [C3 H7 N O]
Sum formula	C85 H99 N21 O33 Zn6 [+ solvent]	C45.50 H56.50 N11.50 O17.50 Zn3
Mr	2335.19	1240.67
Dx,g cm-3	1.377	1.463
Z	1	2
Mu (mm-1)	1.338	1.338
F000	1200.0	1200.0
F000'	1202.20	
h,k,lmax	11,22,22	11,22,22
Nref	10862	10769
Tmin,Tmax	0.676,0.875	0.603,0.745
Tmin'	0.620	

Correction method= # Reported T Limits: Tmin=0.603 Tmax=0.745
AbsCorr = MULTI-SCAN

Data completeness= 0.991 Theta(max)= 25.819

R(reflections)= 0.0577(9205) wR2(reflections)= 0.1782(10769)

S = 1.060 Npar= 601

The following ALERTS were generated. Each ALERT has the format
test-name_ALERT_alert-type_alert-level.
Click on the hyperlinks for more details of the test.

Alert level C

```
PLAT202_ALERT_3_C Isotropic non-H Atoms in Anion/Solvent .........         14 Check
              N10     C39     C40     C41     O15     N9                 etc.
PLAT220_ALERT_2_C NonSolvent   Resd 1  C   Ueq(max)/Ueq(min) Range        5.8 Ratio
PLAT220_ALERT_2_C NonSolvent   Resd 1  N   Ueq(max)/Ueq(min) Range        3.2 Ratio
PLAT220_ALERT_2_C NonSolvent   Resd 1  O   Ueq(max)/Ueq(min) Range        3.2 Ratio
PLAT222_ALERT_3_C NonSolvent Resd 1  H   Uiso(max)/Uiso(min) Range        5.6 Ratio
PLAT241_ALERT_2_C High   'MainMol' Ueq as Compared to Neighbors of         O6 Check
PLAT241_ALERT_2_C High   'MainMol' Ueq as Compared to Neighbors of         O7 Check
PLAT241_ALERT_2_C High   'MainMol' Ueq as Compared to Neighbors of         N5 Check
PLAT241_ALERT_2_C High   'MainMol' Ueq as Compared to Neighbors of         N6 Check
PLAT242_ALERT_2_C Low    'MainMol' Ueq as Compared to Neighbors of        Zn4 Check
PLAT242_ALERT_2_C Low    'MainMol' Ueq as Compared to Neighbors of         N1 Check
PLAT243_ALERT_4_C High   'Solvent' Ueq as Compared to Neighbors of        C37 Check
PLAT243_ALERT_4_C High   'Solvent' Ueq as Compared to Neighbors of        C42 Check
PLAT244_ALERT_4_C Low    'Solvent' Ueq as Compared to Neighbors of        N10 Check
PLAT244_ALERT_4_C Low    'Solvent' Ueq as Compared to Neighbors of        C40 Check
PLAT244_ALERT_4_C Low    'Solvent' Ueq as Compared to Neighbors of         N9 Check
PLAT244_ALERT_4_C Low    'Solvent' Ueq as Compared to Neighbors of        N11 Check
PLAT309_ALERT_2_C Single Bonded Oxygen (C-O > 1.3 Ang) ..........        O16 Check
PLAT317_ALERT_2_C Too many H on C in C=N Moiety in Solvent/Ion ...        C36 Check
PLAT341_ALERT_3_C Low Bond Precision on  C-C Bonds ..............     0.0075 Ang.
PLAT911_ALERT_3_C Missing FCF Refl Between Thmin & STh/L=    0.600         11 Report
PLAT918_ALERT_3_C Reflection(s) with I(obs) much Smaller I(calc) .          3 Check
PLAT971_ALERT_2_C Check Calcd Resid. Dens.  1.54Ang From N11            1.97 eA-3
PLAT971_ALERT_2_C Check Calcd Resid. Dens.  1.09Ang From N3             1.73 eA-3
PLAT975_ALERT_2_C Check Calcd Resid. Dens.  0.94Ang From O17     .      1.32 eA-3
```

Alert level G

```
FORMU01_ALERT_1_G  There is a discrepancy between the atom counts in the
            _chemical_formula_sum and _chemical_formula_moiety. This is
            usually due to the moiety formula being in the wrong format.
            Atom count from _chemical_formula_sum:   C45.5 H56.5 N11.5 O17.5 Zn3
            Atom count from _chemical_formula_moiety:C42.5 H49.5 N10.5 O16.5 Zn3
FORMU01_ALERT_2_G  There is a discrepancy between the atom counts in the
            _chemical_formula_sum and the formula from the _atom_site* data.
            Atom count from _chemical_formula_sum:C45.5 H56.5 N11.5 O17.5 Zn3
            Atom count from the _atom_site data:  C42.5 H49.5 N10.5 O16.5 Zn3
CELLZ01_ALERT_1_G Difference between formula and atom_site contents detected.
CELLZ01_ALERT_1_G ALERT: Large difference may be due to a
            symmetry error - see SYMMG tests
           From the CIF: _cell_formula_units_Z    2
           From the CIF: _chemical_formula_sum  C45.50 H56.50 N11.50 O17.50 Zn3
           TEST: Compare cell contents of formula and atom_site data
```

```
          atom    Z*formula  cif sites diff
          C         91.00     85.00    6.00
          H        113.00     99.00   14.00
          N         23.00     21.00    2.00
          O         35.00     33.00    2.00
          Zn         6.00      6.00    0.00
PLAT002_ALERT_2_G Number of Distance or Angle Restraints on AtSite         12 Note
PLAT003_ALERT_2_G Number of Uiso or Uij Restrained non-H Atoms ...          9 Report
PLAT004_ALERT_5_G Polymeric Structure Found with Maximum Dimension          3 Info
PLAT041_ALERT_1_G Calc. and Reported SumFormula    Strings  Differ     Please Check
PLAT042_ALERT_1_G Calc. and Reported MoietyFormula Strings  Differ     Please Check
PLAT045_ALERT_1_G Calculated and Reported Z Differ by a Factor ...      0.500 Check
PLAT072_ALERT_2_G SHELXL First  Parameter in WGHT  Unusually Large       0.11 Report
PLAT083_ALERT_2_G SHELXL Second Parameter in WGHT  Unusually Large       8.11 Why ?
PLAT171_ALERT_4_G The CIF-Embedded .res File Contains EADP Records          3 Report
PLAT172_ALERT_4_G The CIF-Embedded .res File Contains DFIX Records          4 Report
PLAT187_ALERT_4_G The CIF-Embedded .res File Contains RIGU Records          2 Report
PLAT300_ALERT_4_G Atom Site Occupancy of O16       Constrained at         0.5 Check
PLAT300_ALERT_4_G Atom Site Occupancy of O20       Constrained at         0.5 Check
PLAT300_ALERT_4_G Atom Site Occupancy of H40       Constrained at         0.5 Check
PLAT300_ALERT_4_G Atom Site Occupancy of H40A      Constrained at         0.5 Check
PLAT300_ALERT_4_G Atom Site Occupancy of O18       Constrained at         0.5 Check
PLAT300_ALERT_4_G Atom Site Occupancy of N12       Constrained at         0.5 Check
PLAT300_ALERT_4_G Atom Site Occupancy of C45       Constrained at         0.5 Check
PLAT300_ALERT_4_G Atom Site Occupancy of C46       Constrained at         0.5 Check
PLAT300_ALERT_4_G Atom Site Occupancy of C47       Constrained at         0.5 Check
PLAT300_ALERT_4_G Atom Site Occupancy of H45A      Constrained at         0.5 Check
PLAT300_ALERT_4_G Atom Site Occupancy of H45B      Constrained at         0.5 Check
PLAT300_ALERT_4_G Atom Site Occupancy of H45C      Constrained at         0.5 Check
PLAT300_ALERT_4_G Atom Site Occupancy of H46       Constrained at         0.5 Check
PLAT300_ALERT_4_G Atom Site Occupancy of H47A      Constrained at         0.5 Check
PLAT300_ALERT_4_G Atom Site Occupancy of H47B      Constrained at         0.5 Check
PLAT300_ALERT_4_G Atom Site Occupancy of H47C      Constrained at         0.5 Check
PLAT301_ALERT_3_G Main Residue  Disorder .............(Resd  1  )          7% Note
PLAT302_ALERT_4_G Anion/Solvent/Minor-Residue Disorder (Resd  2  )        20% Note
PLAT302_ALERT_4_G Anion/Solvent/Minor-Residue Disorder (Resd  5  )       100% Note
PLAT412_ALERT_2_G Short Intra XH3 .. XHn     H40A     ..H41B     .       2.09 Ang.
                                                    x,y,z  =      1_555 Check
PLAT413_ALERT_2_G Short Inter XH3 .. XHn     H38C     ..H35C     .       2.04 Ang.
                                              3-x,2-y,1-z  =      2_876 Check
PLAT605_ALERT_4_G Largest Solvent Accessible VOID in the Structure        282 A**3
PLAT789_ALERT_4_G Atoms with Negative _atom_site_disorder_group  #         12 Check
PLAT794_ALERT_5_G Tentative Bond Valency for Zn1       (II)      .       2.14 Info
PLAT794_ALERT_5_G Tentative Bond Valency for Zn2       (II)      .       2.11 Info
PLAT794_ALERT_5_G Tentative Bond Valency for Zn3       (II)      .       2.04 Info
PLAT860_ALERT_3_G Number of Least-Squares Restraints ............          51 Note
PLAT910_ALERT_3_G Missing # of FCF Reflection(s) Below Theta(Min).          3 Note
PLAT912_ALERT_4_G Missing # of FCF Reflections Above STh/L=  0.600         80 Note
PLAT933_ALERT_2_G Number of HKL-OMIT Records in Embedded .res File          6 Note
PLAT941_ALERT_3_G Average HKL Measurement Multiplicity ..........         3.5 Low
PLAT978_ALERT_2_G Number C-C Bonds with Positive Residual Density.          0 Info
```

---

0 **ALERT level A** = Most likely a serious problem - resolve or explain
0 **ALERT level B** = A potentially serious problem, consider carefully
25 **ALERT level C** = Check. Ensure it is not caused by an omission or oversight
47 **ALERT level G** = General information/check it is not something unexpected

```
 6 ALERT type 1 CIF construction/syntax error, inconsistent or missing data
23 ALERT type 2 Indicator that the structure model may be wrong or deficient
 9 ALERT type 3 Indicator that the structure quality may be low
30 ALERT type 4 Improvement, methodology, query or suggestion
 4 ALERT type 5 Informative message, check
```

It is advisable to attempt to resolve as many as possible of the alerts in all categories. Often the minor alerts point to easily fixed oversights, errors and omissions in your CIF or refinement strategy, so attention to these fine details can be worthwhile. In order to resolve some of the more serious problems it may be necessary to carry out additional measurements or structure refinements. However, the purpose of your study may justify the reported deviations and the more serious of these should normally be commented upon in the discussion or experimental section of a paper or in the "special_details" fields of the CIF. checkCIF was carefully designed to identify outliers and unusual parameters, but every test has its limitations and alerts that are not important in a particular case may appear. Conversely, the absence of alerts does not guarantee there are no aspects of the results needing attention. It is up to the individual to critically assess their own results and, if necessary, seek expert advice.

Publication of your CIF in IUCr journals

A basic structural check has been run on your CIF. These basic checks will be run on all CIFs submitted for publication in IUCr journals (*Acta Crystallographica*, *Journal of Applied Crystallography*, *Journal of Synchrotron Radiation*); however, if you intend to submit to *Acta Crystallographica Section C* or *E* or *IUCrData*, you should make sure that full publication checks are run on the final version of your CIF prior to submission.

Publication of your CIF in other journals

Please refer to the *Notes for Authors* of the relevant journal for any special instructions relating to CIF submission.

PLATON version of 12/09/2022; check.def file version of 09/08/2022

Datablock tgs125 - ellipsoid plot

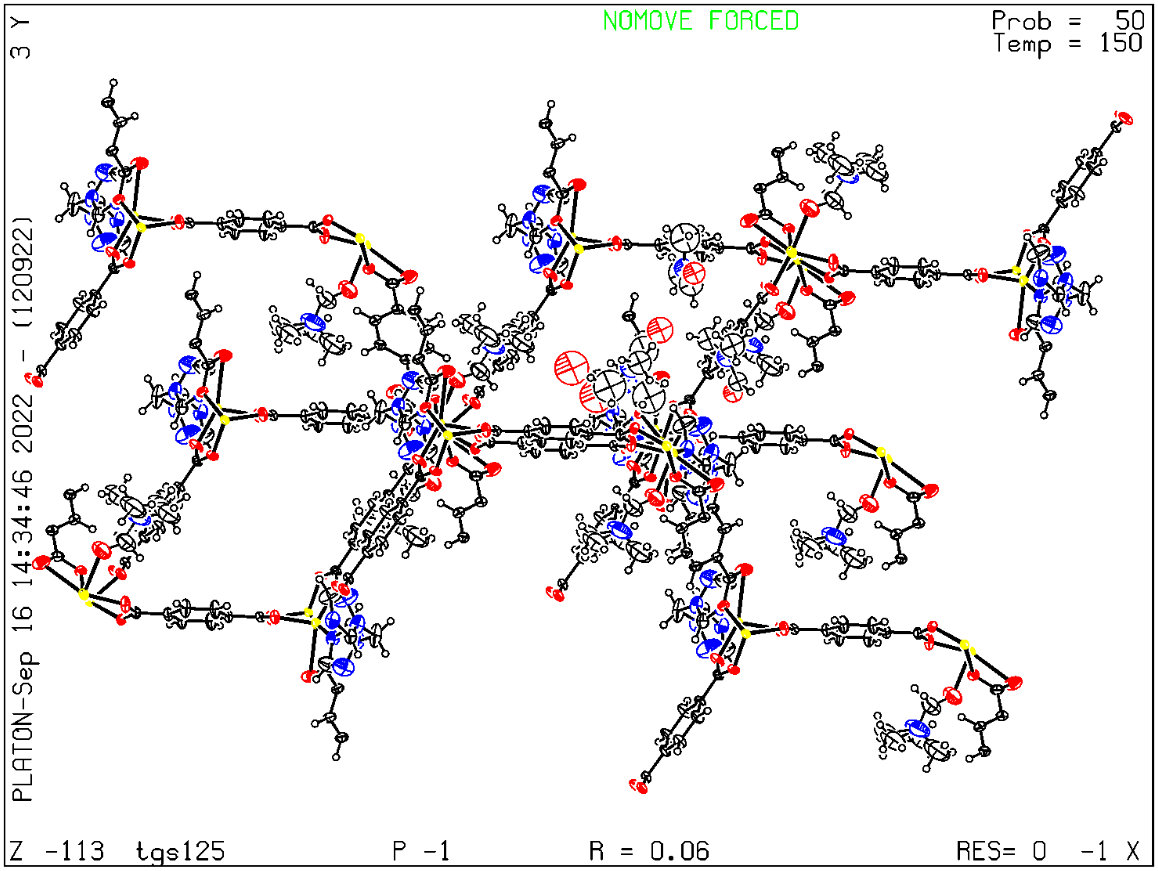

Supplement: Supplementary file 1 [file molecules-27-06481-s001.zip › 1.pdf]
