# Supplementary material for: Structural Diversity and Carbon Dioxide Sorption Selectivity of Zinc(II) Metal-Organic Frameworks Based on Bis(1,2,4-triazol-1-yl)methane and Terephthalic Acid
Source: Molecules. 2022 Oct 1;27(19):6481. doi: 10.3390/molecules27196481 (PMC9571910; doi:10.3390/molecules27196481)

checkCIF/PLATON report

Structure factors have been supplied for datablock(s) tgs142

THIS REPORT IS FOR GUIDANCE ONLY. IF USED AS PART OF A REVIEW PROCEDURE FOR PUBLICATION, IT SHOULD NOT REPLACE THE EXPERTISE OF AN EXPERIENCED CRYSTALLOGRAPHIC REFEREE.

No syntax errors found. CIF dictionary Interpreting this report

Datablock: tgs142

Bond precision:	C-C = 0.0107 A		Wavelength=0.71073
Cell:	a=20.1879(7)	b=18.2491(6)	c=32.9335(10)
	alpha=90	beta=90	gamma=90
Temperature:	298 K		

	Calculated	Reported
Volume	12133.1(7)	12133.1(7)
Space group	P b c a	P b c a
Hall group	-P 2ac 2ab	-P 2ac 2ab
Moiety formula	C29 H18 N6 O12 Zn3 [+ solvent]	C29 H18 N6 O12 Zn3, 8[C3 H7 N O]
Sum formula	C29 H18 N6 O12 Zn3 [+ solvent]	C53 H74 N14 O20 Zn3
Mr	838.66	1423.40
Dx,g cm-3	0.918	1.558
Z	8	8
Mu (mm-1)	1.215	1.215
F000	3360.0	3360.0
F000'	3368.22	
h,k,lmax	23,21,38	23,21,38
Nref	9988	9982
Tmin,Tmax	0.864,0.886	0.570,0.745
Tmin'	0.784	

Correction method= # Reported T Limits: Tmin=0.570 Tmax=0.745
AbsCorr = MULTI-SCAN

Data completeness= 0.999 Theta(max)= 24.405

R(reflections)= 0.0788(8234)
wR2(reflections)= 0.2172(9982)

S = 1.059
Npar= 451

The following ALERTS were generated. Each ALERT has the format
test-name_ALERT_alert-type_alert-level.
Click on the hyperlinks for more details of the test.

Alert level C

ABSMU01_ALERT_1_C The ratio of given/expected absorption coefficient lies
outside the range 0.99 <> 1.01
Calculated value of mu = 1.263
Value of mu given = 1.215
THETM01_ALERT_3_C The value of sine(theta_max)/wavelength is less than 0.590
Calculated sin(theta_max)/wavelength = 0.5814
PLAT213_ALERT_2_C Atom C28 has ADP max/min Ratio ..... 3.4 prolat
PLAT220_ALERT_2_C NonSolvent Resd 1 C Ueq(max)/Ueq(min) Range 3.5 Ratio
PLAT241_ALERT_2_C High 'MainMol' Ueq as Compared to Neighbors of O5 Check
PLAT241_ALERT_2_C High 'MainMol' Ueq as Compared to Neighbors of O6 Check
PLAT241_ALERT_2_C High 'MainMol' Ueq as Compared to Neighbors of O7 Check
PLAT241_ALERT_2_C High 'MainMol' Ueq as Compared to Neighbors of O8 Check
PLAT241_ALERT_2_C High 'MainMol' Ueq as Compared to Neighbors of O9 Check
PLAT241_ALERT_2_C High 'MainMol' Ueq as Compared to Neighbors of O10 Check
PLAT241_ALERT_2_C High 'MainMol' Ueq as Compared to Neighbors of N4 Check
PLAT241_ALERT_2_C High 'MainMol' Ueq as Compared to Neighbors of N6 Check
PLAT241_ALERT_2_C High 'MainMol' Ueq as Compared to Neighbors of C26 Check
PLAT241_ALERT_2_C High 'MainMol' Ueq as Compared to Neighbors of C28 Check
PLAT241_ALERT_2_C High 'MainMol' Ueq as Compared to Neighbors of C29 Check
PLAT242_ALERT_2_C Low 'MainMol' Ueq as Compared to Neighbors of Zn1 Check
PLAT242_ALERT_2_C Low 'MainMol' Ueq as Compared to Neighbors of Zn2 Check
PLAT242_ALERT_2_C Low 'MainMol' Ueq as Compared to Neighbors of Zn3 Check
PLAT242_ALERT_2_C Low 'MainMol' Ueq as Compared to Neighbors of N1 Check
PLAT242_ALERT_2_C Low 'MainMol' Ueq as Compared to Neighbors of N2 Check
PLAT242_ALERT_2_C Low 'MainMol' Ueq as Compared to Neighbors of N3 Check
PLAT242_ALERT_2_C Low 'MainMol' Ueq as Compared to Neighbors of C11 Check
PLAT334_ALERT_2_C Small <C-C> Benzene Dist. C7 -C24 . 1.37 Ang.
PLAT341_ALERT_3_C Low Bond Precision on C-C Bonds .... 0.01067 Ang.
PLAT911_ALERT_3_C Missing FCF Refl Between Thmin & STh/L= 0.581 7 Report
PLAT918_ALERT_3_C Reflection(s) with I(obs) much Smaller I(calc) . 4 Check
PLAT975_ALERT_2_C Check Calcd Resid. Dens. 1.08Ang From O3 . 0.58 eA-3
PLAT975_ALERT_2_C Check Calcd Resid. Dens. 0.92Ang From O8 . 0.56 eA-3
PLAT977_ALERT_2_C Check Negative Difference Density on H21 . -0.31 eA-3

Alert level G

FORMU01_ALERT_2_G There is a discrepancy between the atom counts in the
_chemical_formula_sum and the formula from the _atom_site* data.
Atom count from _chemical_formula_sum:C53 H74 N14 O20 Zn3
Atom count from the _atom_site data: C29 H18 N6 O12 Zn3
CELLZ01_ALERT_1_G Difference between formula and atom_site contents detected.
CELLZ01_ALERT_1_G ALERT: Large difference may be due to a
symmetry error - see SYMMG tests
From the CIF: _cell_formula_units_Z 8
From the CIF: _chemical_formula_sum C53 H74 N14 O20 Zn3

TEST: Compare cell contents of formula and atom_site data

atom	Z*formula	cif sites	diff
C	424.00	232.00	192.00
H	592.00	144.00	448.00
N	112.00	48.00	64.00
O	160.00	96.00	64.00
Zn	24.00	24.00	0.00

```
PLAT003_ALERT_2_G Number of Uiso or Uij Restrained non-H Atoms ...          11 Report
PLAT004_ALERT_5_G Polymeric Structure Found with Maximum Dimension           3 Info
PLAT041_ALERT_1_G Calc. and Reported SumFormula    Strings  Differ     Please Check
PLAT083_ALERT_2_G SHELXL Second Parameter in WGHT  Unusually Large      88.50 Why ?
PLAT187_ALERT_4_G The CIF-Embedded .res File Contains RIGU Records           1 Report
PLAT606_ALERT_4_G Solvent Accessible VOID(S) in Structure ........           ! Info
PLAT794_ALERT_5_G Tentative Bond Valency for Zn1       (II)      .        2.19 Info
PLAT794_ALERT_5_G Tentative Bond Valency for Zn2       (II)      .        2.07 Info
PLAT794_ALERT_5_G Tentative Bond Valency for Zn3       (II)      .        2.19 Info
PLAT860_ALERT_3_G Number of Least-Squares Restraints ............          81 Note
PLAT909_ALERT_3_G Percentage of I>2sig(I) Data at Theta(Max) Still        60% Note
PLAT910_ALERT_3_G Missing # of FCF Reflection(s) Below Theta(Min).           1 Note
PLAT913_ALERT_3_G Missing # of Very Strong Reflections in FCF ....           1 Note
PLAT933_ALERT_2_G Number of HKL-OMIT Records in Embedded .res File           2 Note
PLAT978_ALERT_2_G Number C-C Bonds with Positive Residual Density.           0 Info
```

---

0 **ALERT level A** = Most likely a serious problem - resolve or explain
0 **ALERT level B** = A potentially serious problem, consider carefully
29 **ALERT level C** = Check. Ensure it is not caused by an omission or oversight
18 **ALERT level G** = General information/check it is not something unexpected

4 ALERT type 1 CIF construction/syntax error, inconsistent or missing data
29 ALERT type 2 Indicator that the structure model may be wrong or deficient
8 ALERT type 3 Indicator that the structure quality may be low
2 ALERT type 4 Improvement, methodology, query or suggestion
4 ALERT type 5 Informative message, check

It is advisable to attempt to resolve as many as possible of the alerts in all categories. Often the minor alerts point to easily fixed oversights, errors and omissions in your CIF or refinement strategy, so attention to these fine details can be worthwhile. In order to resolve some of the more serious problems it may be necessary to carry out additional measurements or structure refinements. However, the purpose of your study may justify the reported deviations and the more serious of these should normally be commented upon in the discussion or experimental section of a paper or in the "special_details" fields of the CIF. checkCIF was carefully designed to identify outliers and unusual parameters, but every test has its limitations and alerts that are not important in a particular case may appear. Conversely, the absence of alerts does not guarantee there are no aspects of the results needing attention. It is up to the individual to critically assess their own results and, if necessary, seek expert advice.

Publication of your CIF in IUCr journals

A basic structural check has been run on your CIF. These basic checks will be run on all CIFs submitted for publication in IUCr journals (*Acta Crystallographica*, *Journal of Applied Crystallography*, *Journal of Synchrotron Radiation*); however, if you intend to submit to *Acta Crystallographica Section C* or *E* or *IUCrData*, you should make sure that full publication checks are run on the final version of your CIF prior to submission.

Publication of your CIF in other journals

Please refer to the *Notes for Authors* of the relevant journal for any special instructions relating to CIF submission.

PLATON version of 12/09/2022; check.def file version of 09/08/2022

Datablock tgs142 - ellipsoid plot

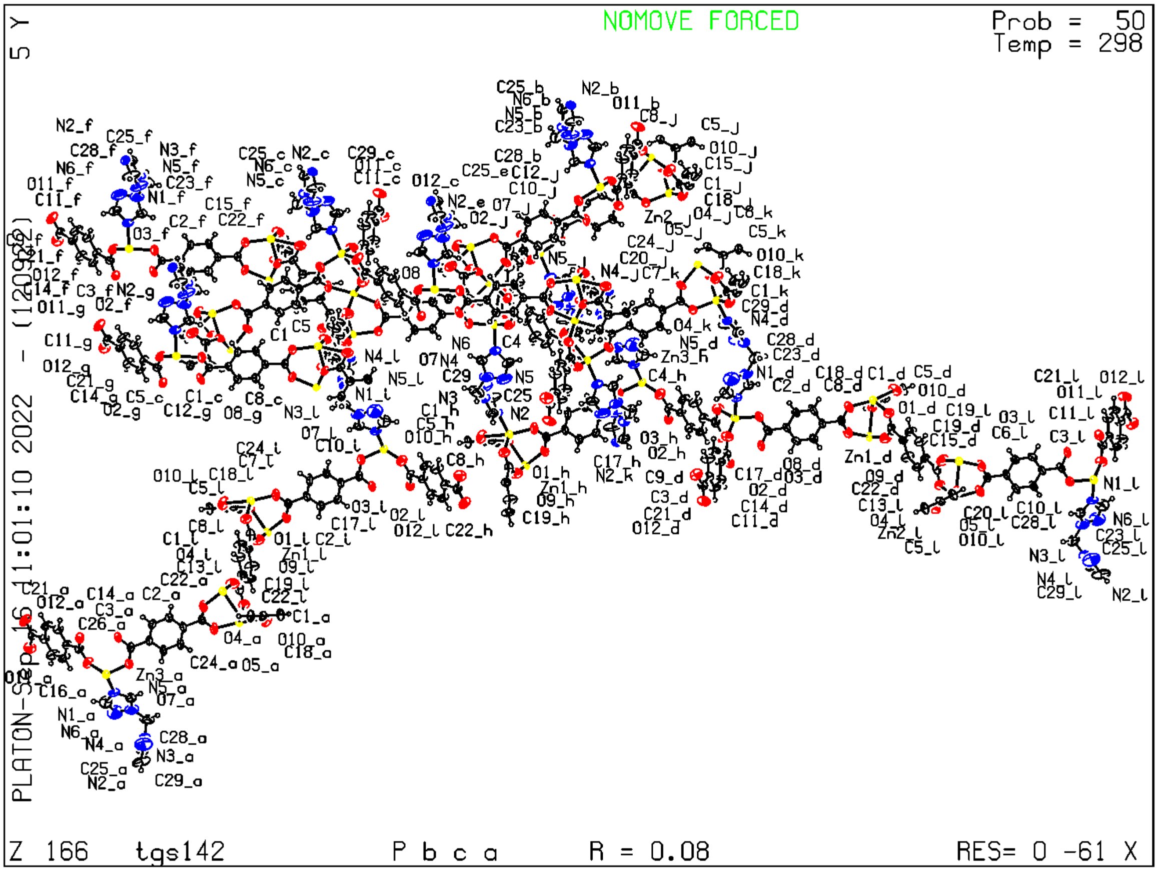

Supplement: Supplementary file 1 [file molecules-27-06481-s001.zip › 2.pdf]
