# Supplementary material for: Structural Diversity and Carbon Dioxide Sorption Selectivity of Zinc(II) Metal-Organic Frameworks Based on Bis(1,2,4-triazol-1-yl)methane and Terephthalic Acid
Source: Molecules. 2022 Oct 1;27(19):6481. doi: 10.3390/molecules27196481 (PMC9571910; doi:10.3390/molecules27196481)

checkCIF/PLATON report

Structure factors have been supplied for datablock(s) tgs232_1

THIS REPORT IS FOR GUIDANCE ONLY. IF USED AS PART OF A REVIEW PROCEDURE FOR PUBLICATION, IT SHOULD NOT REPLACE THE EXPERTISE OF AN EXPERIENCED CRYSTALLOGRAPHIC REFEREE.

No syntax errors found. CIF dictionary Interpreting this report

Datablock: tgs232_1

```
Bond precision:   C-C = 0.0101 A          Wavelength=0.71073

Cell:              a=23.893(3)     b=16.604(3)          c=17.746(3)
                   alpha=90        beta=124.479(7)      gamma=90
Temperature:       298 K
```

	Calculated	Reported
Volume	5803.5(17)	5803.7(14)
Space group	C 2/c	C 1 2/c 1
Hall group	-C 2yc	-C 2yc
Moiety formula	C13 H10 N6 O4 Zn [+ solvent]	C13 H10 N6 O4 Zn, 3[C3 H7 N O]
Sum formula	C13 H10 N6 O4 Zn [+ solvent]	C22 H31 N9 O7 Zn
Mr	379.66	598.90
Dx,g cm-3	0.869	1.371
Z	8	8
Mu (mm-1)	0.863	0.863
F000	1536.0	1536.0
F000'	1538.81	
h,k,lmax	27,19,20	27,19,20
Nref	4779	4771
Tmin,Tmax	0.813,0.917	0.635,0.745
Tmin'	0.772	

```
Correction method= # Reported T Limits: Tmin=0.635 Tmax=0.745
AbsCorr = MULTI-SCAN

Data completeness= 0.998            Theta(max)= 24.403
```

R(reflections)= 0.0668(3441)

wR2(reflections)= 0.2197(4771)

S = 1.032 Npar= 239

The following ALERTS were generated. Each ALERT has the format
test-name_ALERT_alert-type_alert-level.
Click on the hyperlinks for more details of the test.

Alert level C

ABSMU01_ALERT_1_C The ratio of given/expected absorption coefficient lies outside the range 0.99 <> 1.01
Calculated value of mu = 0.901
Value of mu given = 0.863
THETM01_ALERT_3_C The value of sine(theta_max)/wavelength is less than 0.590
Calculated sin(theta_max)/wavelength = 0.5813
PLAT213_ALERT_2_C Atom O5 has ADP max/min Ratio ..... 3.3 oblate
PLAT234_ALERT_4_C Large Hirshfeld Difference Zn1 --O3_d . 0.18 Ang.
PLAT234_ALERT_4_C Large Hirshfeld Difference Zn1 --O4_d . 0.16 Ang.
PLAT241_ALERT_2_C High 'MainMol' Ueq as Compared to Neighbors of N6 Check
PLAT241_ALERT_2_C High 'MainMol' Ueq as Compared to Neighbors of C12 Check
PLAT242_ALERT_2_C Low 'MainMol' Ueq as Compared to Neighbors of C4 Check
PLAT250_ALERT_2_C Large U3/U1 Ratio for Average U(i,j) Tensor .... 2.5 Note
PLAT334_ALERT_2_C Small <C-C> Benzene Dist. C4 -C10 . 1.37 Ang.
PLAT341_ALERT_3_C Low Bond Precision on C-C Bonds .... 0.01014 Ang.
PLAT905_ALERT_3_C Negative K value in the Analysis of Variance ... -10.486 Report
PLAT911_ALERT_3_C Missing FCF Refl Between Thmin & STh/L= 0.581 9 Report
PLAT918_ALERT_3_C Reflection(s) with I(obs) much Smaller I(calc) . 1 Check

---

Alert level G

FORMU01_ALERT_2_G There is a discrepancy between the atom counts in the _chemical_formula_sum and the formula from the _atom_site* data.
Atom count from _chemical_formula_sum:C22 H31 N9 O7 Zn1
Atom count from the _atom_site data: C13 H10 N6 O4 Zn1
CELLZ01_ALERT_1_G Difference between formula and atom_site contents detected.
CELLZ01_ALERT_1_G ALERT: Large difference may be due to a symmetry error - see SYMMG tests
From the CIF: _cell_formula_units_Z 8
From the CIF: _chemical_formula_sum C22 H31 N9 O7 Zn
TEST: Compare cell contents of formula and atom_site data

atom	Z*formula	cif sites	diff
C	176.00	104.00	72.00
H	248.00	80.00	168.00
N	72.00	48.00	24.00
O	56.00	32.00	24.00
Zn	8.00	8.00	0.00

PLAT002_ALERT_2_G Number of Distance or Angle Restraints on AtSite 6 Note
PLAT003_ALERT_2_G Number of Uiso or Uij Restrained non-H Atoms ... 4 Report
PLAT004_ALERT_5_G Polymeric Structure Found with Maximum Dimension 3 Info
PLAT041_ALERT_1_G Calc. and Reported SumFormula Strings Differ Please Check
PLAT072_ALERT_2_G SHELXL First Parameter in WGHT Unusually Large 0.14 Report
PLAT083_ALERT_2_G SHELXL Second Parameter in WGHT Unusually Large 10.06 Why ?
PLAT128_ALERT_4_G Alternate Setting for Input Space Group C2/c I2/a Note

```
PLAT152_ALERT_1_G The Supplied and Calc. Volume s.u. Differ by ...          3 Units
PLAT171_ALERT_4_G The CIF-Embedded .res File Contains EADP Records          1 Report
PLAT176_ALERT_4_G The CIF-Embedded .res File Contains SADI Records          1 Report
PLAT187_ALERT_4_G The CIF-Embedded .res File Contains RIGU Records          1 Report
PLAT301_ALERT_3_G Main Residue  Disorder .............(Resd  1  )         13% Note
PLAT432_ALERT_2_G Short Inter X...Y Contact  O3       ..C3       .       2.70 Ang.
                                               1-x,y,3/2-z  =      2_656 Check
PLAT606_ALERT_4_G Solvent Accessible VOID(S) in Structure ........          ! Info
PLAT860_ALERT_3_G Number of Least-Squares Restraints ............          24 Note
PLAT909_ALERT_3_G Percentage of I>2sig(I) Data at Theta(Max) Still        46% Note
PLAT933_ALERT_2_G Number of HKL-OMIT Records in Embedded .res File          5 Note
PLAT978_ALERT_2_G Number C-C Bonds with Positive Residual Density.          0 Info
```

```
   0 ALERT level A = Most likely a serious problem - resolve or explain
   0 ALERT level B = A potentially serious problem, consider carefully
  14 ALERT level C = Check. Ensure it is not caused by an omission or oversight
  21 ALERT level G = General information/check it is not something unexpected

   5 ALERT type 1 CIF construction/syntax error, inconsistent or missing data
  14 ALERT type 2 Indicator that the structure model may be wrong or deficient
   8 ALERT type 3 Indicator that the structure quality may be low
   7 ALERT type 4 Improvement, methodology, query or suggestion
   1 ALERT type 5 Informative message, check
```

It is advisable to attempt to resolve as many as possible of the alerts in all categories. Often the minor alerts point to easily fixed oversights, errors and omissions in your CIF or refinement strategy, so attention to these fine details can be worthwhile. In order to resolve some of the more serious problems it may be necessary to carry out additional measurements or structure refinements. However, the purpose of your study may justify the reported deviations and the more serious of these should normally be commented upon in the discussion or experimental section of a paper or in the "special_details" fields of the CIF. checkCIF was carefully designed to identify outliers and unusual parameters, but every test has its limitations and alerts that are not important in a particular case may appear. Conversely, the absence of alerts does not guarantee there are no aspects of the results needing attention. It is up to the individual to critically assess their own results and, if necessary, seek expert advice.

Publication of your CIF in IUCr journals

A basic structural check has been run on your CIF. These basic checks will be run on all CIFs submitted for publication in IUCr journals (*Acta Crystallographica*, *Journal of Applied Crystallography*, *Journal of Synchrotron Radiation*); however, if you intend to submit to *Acta Crystallographica Section C* or *E* or *IUCrData*, you should make sure that full publication checks are run on the final version of your CIF prior to submission.

Publication of your CIF in other journals

Please refer to the *Notes for Authors* of the relevant journal for any special instructions relating to CIF submission.

PLATON version of 12/09/2022; check.def file version of 09/08/2022

Datablock tgs232_1 - ellipsoid plot

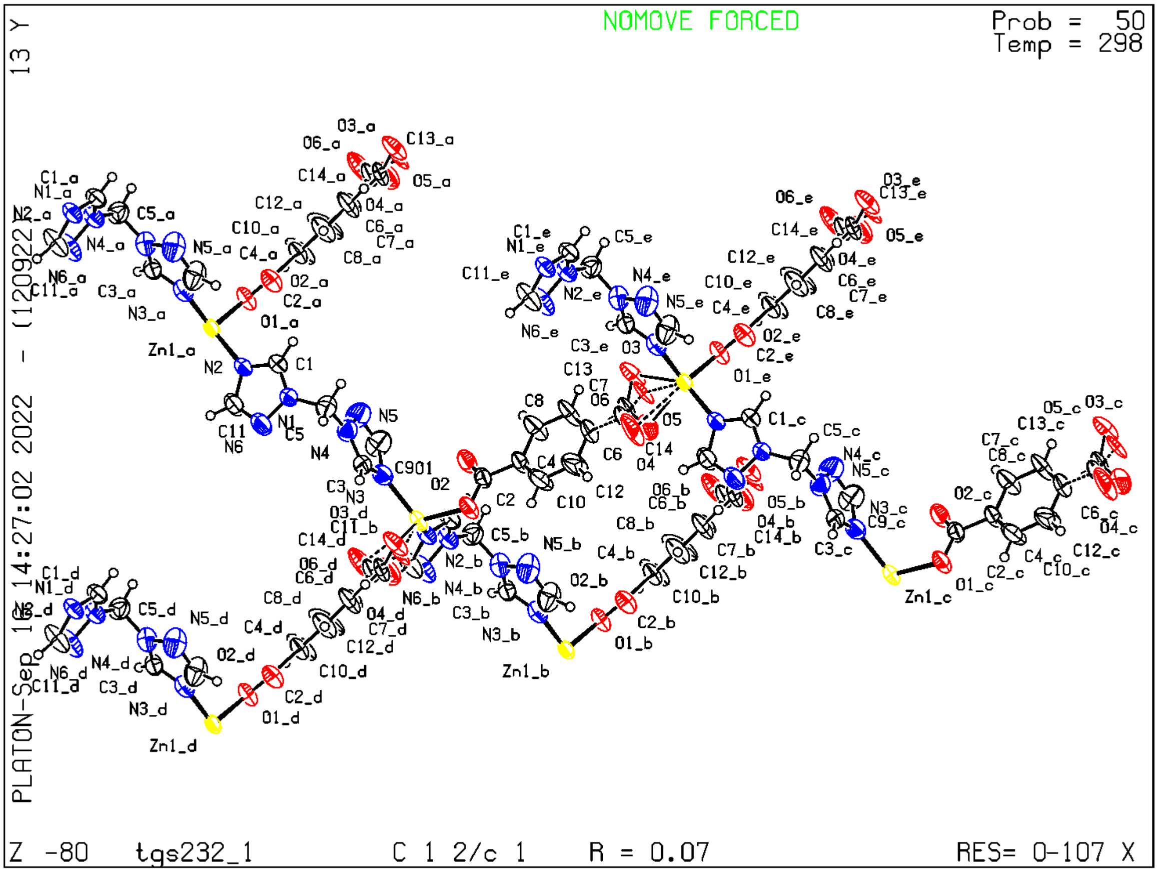

Supplement: Supplementary file 1 [file molecules-27-06481-s001.zip › 3.pdf]
