# Supplementary material for: Structural Diversity and Carbon Dioxide Sorption Selectivity of Zinc(II) Metal-Organic Frameworks Based on Bis(1,2,4-triazol-1-yl)methane and Terephthalic Acid
Source: Molecules. 2022 Oct 1;27(19):6481. doi: 10.3390/molecules27196481 (PMC9571910; doi:10.3390/molecules27196481)

checkCIF/PLATON report

Structure factors have been supplied for datablock(s) tgs142_a_1

THIS REPORT IS FOR GUIDANCE ONLY. IF USED AS PART OF A REVIEW PROCEDURE FOR PUBLICATION, IT SHOULD NOT REPLACE THE EXPERTISE OF AN EXPERIENCED CRYSTALLOGRAPHIC REFEREE.

No syntax errors found. CIF dictionary Interpreting this report

Datablock: tgs142_a_1

Bond precision:	C-C = 0.0064 A		Wavelength=0.71073
Cell:	a=19.9831(15)	b=16.6095(13)	c=17.4898(13)
	alpha=90	beta=102.142(2)	gamma=90
Temperature:	150 K		

	Calculated	Reported
Volume	5675.2(7)	5675.2(7)
Space group	P 21/c	P 1 21/c 1
Hall group	-P 2ybc	-P 2ybc
Moiety formula	C26 H20 N12 O8 Zn2, 6(C3 H7 N O)	0.25(C52 H40 N24 O16 Zn4), 3(C3 H7 N O)
Sum formula	C44 H62 N18 O14 Zn2	C22 H31 N9 O7 Zn
Mr	1197.90	598.93
Dx,g cm-3	1.402	1.402
Z	4	8
Mu (mm-1)	0.921	0.921
F000	2496.0	2496.0
F000'	2499.22	
h,k,lmax	23,19,20	23,19,20
Nref	9347	9328
Tmin,Tmax	0.847,0.929	0.651,0.745
Tmin'	0.787	

Correction method= # Reported T Limits: Tmin=0.651 Tmax=0.745
AbsCorr = MULTI-SCAN

Data completeness= 0.998 Theta(max)= 24.406

R(reflections)= 0.0506(5414) wR2(reflections)= 0.1681(9328)

S = 1.060 Npar= 715

The following ALERTS were generated. Each ALERT has the format
test-name_ALERT_alert-type_alert-level.
Click on the hyperlinks for more details of the test.

Alert level C

```
THETM01_ALERT_3_C  The value of sine(theta_max)/wavelength is less than 0.590
            Calculated sin(theta_max)/wavelength =    0.5814
PLAT042_ALERT_1_C Calc. and Reported MoietyFormula Strings  Differ     Please Check
PLAT244_ALERT_4_C Low    'Solvent' Ueq as Compared to Neighbors of        N18 Check
PLAT244_ALERT_4_C Low    'Solvent' Ueq as Compared to Neighbors of        N15 Check
PLAT244_ALERT_4_C Low    'Solvent' Ueq as Compared to Neighbors of        N16 Check
PLAT244_ALERT_4_C Low    'Solvent' Ueq as Compared to Neighbors of        N14 Check
PLAT244_ALERT_4_C Low    'Solvent' Ueq as Compared to Neighbors of        N13 Check
PLAT244_ALERT_4_C Low    'Solvent' Ueq as Compared to Neighbors of        N17 Check
PLAT250_ALERT_2_C Large U3/U1 Ratio for Average U(i,j) Tensor ....        2.3 Note
PLAT341_ALERT_3_C Low Bond Precision on  C-C Bonds ..............    0.00644 Ang.
PLAT905_ALERT_3_C Negative K value in the Analysis of Variance ...     -8.404 Report
PLAT905_ALERT_3_C Negative K value in the Analysis of Variance ...     -0.614 Report
PLAT911_ALERT_3_C Missing FCF Refl Between Thmin & STh/L=    0.581         20 Report
PLAT918_ALERT_3_C Reflection(s) with I(obs) much Smaller I(calc) .          1 Check
```

Alert level G

```
PLAT004_ALERT_5_G Polymeric Structure Found with Maximum Dimension          3 Info
PLAT045_ALERT_1_G Calculated and Reported Z Differ by a Factor ...      0.500 Check
PLAT083_ALERT_2_G SHELXL Second Parameter in WGHT  Unusually Large      11.53 Why ?
PLAT794_ALERT_5_G Tentative Bond Valency for Zn1       (II)      .       1.89 Info
PLAT794_ALERT_5_G Tentative Bond Valency for Zn2       (II)      .       1.86 Info
PLAT909_ALERT_3_G Percentage of I>2sig(I) Data at Theta(Max) Still        33% Note
PLAT933_ALERT_2_G Number of HKL-OMIT Records in Embedded .res File          3 Note
PLAT978_ALERT_2_G Number C-C Bonds with Positive Residual Density.          0 Info
```

0 **ALERT level A** = Most likely a serious problem - resolve or explain
0 **ALERT level B** = A potentially serious problem, consider carefully
14 **ALERT level C** = Check. Ensure it is not caused by an omission or oversight
8 **ALERT level G** = General information/check it is not something unexpected

2 ALERT type 1 CIF construction/syntax error, inconsistent or missing data
4 ALERT type 2 Indicator that the structure model may be wrong or deficient
7 ALERT type 3 Indicator that the structure quality may be low
6 ALERT type 4 Improvement, methodology, query or suggestion
3 ALERT type 5 Informative message, check

It is advisable to attempt to resolve as many as possible of the alerts in all categories. Often the minor alerts point to easily fixed oversights, errors and omissions in your CIF or refinement strategy, so attention to these fine details can be worthwhile. In order to resolve some of the more serious problems it may be necessary to carry out additional measurements or structure refinements. However, the purpose of your study may justify the reported deviations and the more serious of these should normally be commented upon in the discussion or experimental section of a paper or in the "special_details" fields of the CIF. checkCIF was carefully designed to identify outliers and unusual parameters, but every test has its limitations and alerts that are not important in a particular case may appear. Conversely, the absence of alerts does not guarantee there are no aspects of the results needing attention. It is up to the individual to critically assess their own results and, if necessary, seek expert advice.

Publication of your CIF in IUCr journals

A basic structural check has been run on your CIF. These basic checks will be run on all CIFs submitted for publication in IUCr journals (*Acta Crystallographica*, *Journal of Applied Crystallography*, *Journal of Synchrotron Radiation*); however, if you intend to submit to *Acta Crystallographica Section C* or *E* or *IUCrData*, you should make sure that full publication checks are run on the final version of your CIF prior to submission.

Publication of your CIF in other journals

Please refer to the *Notes for Authors* of the relevant journal for any special instructions relating to CIF submission.

PLATON version of 12/09/2022; check.def file version of 09/08/2022

Datablock tgs142_a_1 - ellipsoid plot

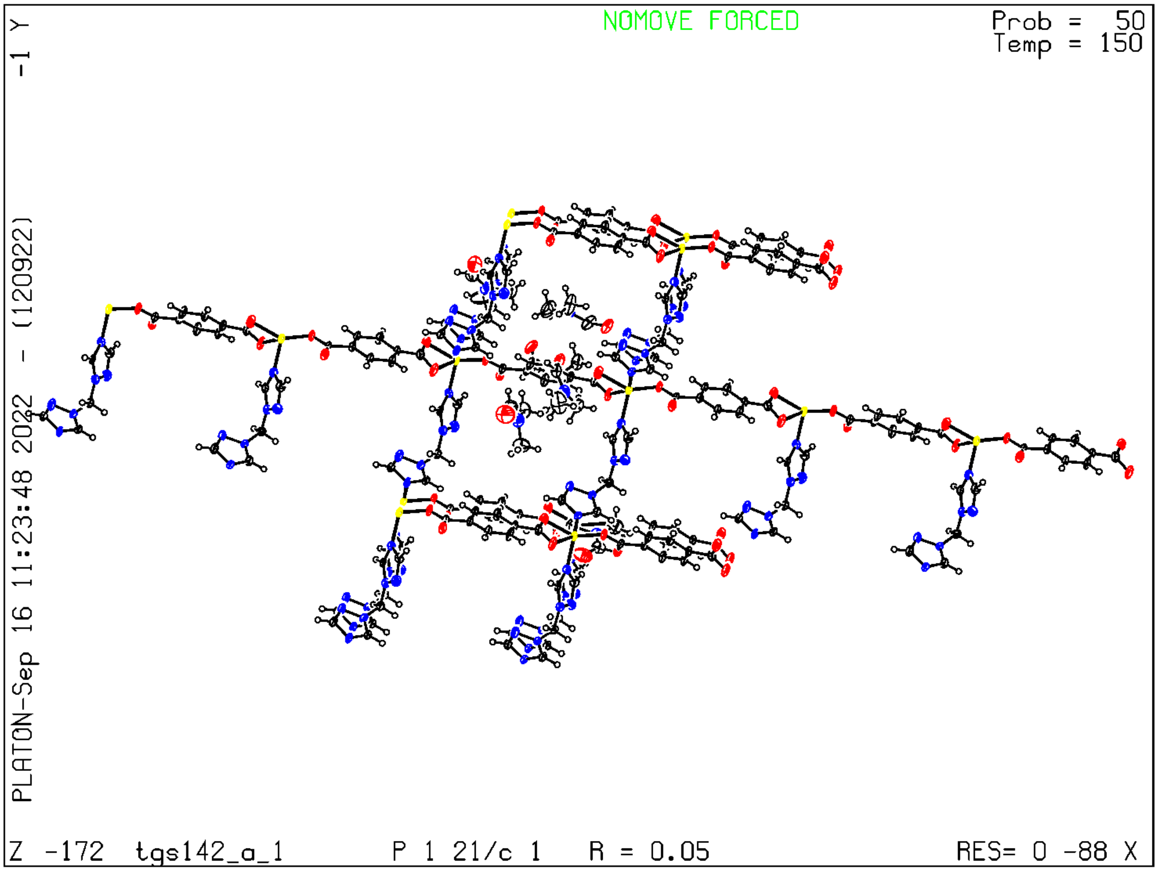

Supplement: Supplementary file 1 [file molecules-27-06481-s001.zip › 3a.pdf]
