# Supplementary material for: Structural Diversity and Carbon Dioxide Sorption Selectivity of Zinc(II) Metal-Organic Frameworks Based on Bis(1,2,4-triazol-1-yl)methane and Terephthalic Acid
Source: Molecules. 2022 Oct 1;27(19):6481. doi: 10.3390/molecules27196481 (PMC9571910; doi:10.3390/molecules27196481)
Supplement: Supplementary file 1 [file molecules-27-06481-s001.zip › Supplementary Materials.pdf]

Supplementary Materials

Structural diversity and carbon dioxide sorption selectivity of zinc(II) metal-organic frameworks based on bis(1,2,4-triazol-1-yl)methane and terephthalic acid

Taisiya S. Sukhikh [1], Evgeny Yu. Filatov [1], Alexey A. Ryadun [1], Konstantin A. Kovalenko [1] and Andrei S. Potapov [1,*]

[1] Nikolaev Institute of Inorganic Chemistry, Siberian Branch of the Russian Academy of Sciences, 3 Lavrentiev Ave., 630090 Novosibirsk, Russia

* Correspondence: potapov@niic.nsc.ru; Tel.: +7 (383) 330-94-90

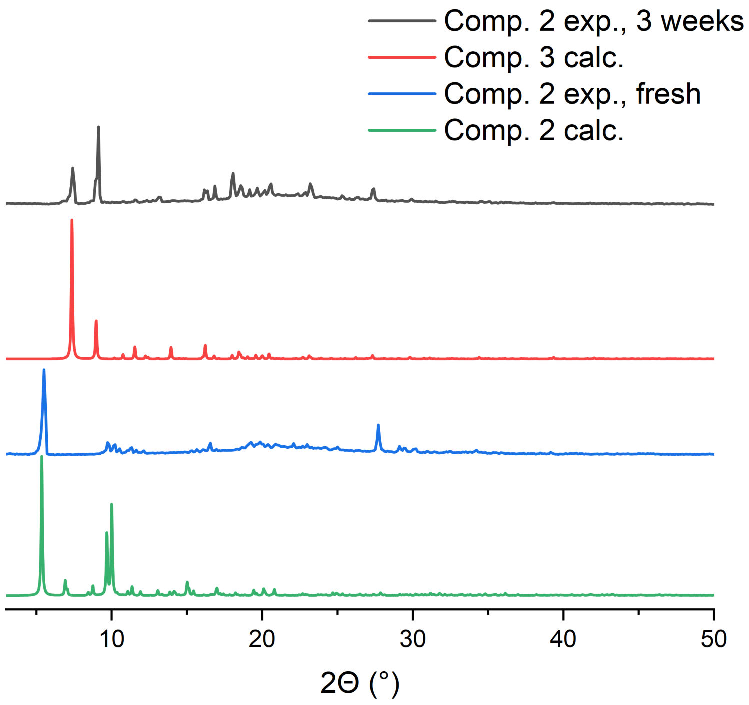

Figure S1. Calculated and experimental XRD patterns of MOF **2** and the product of its transformation after storing under DMF.

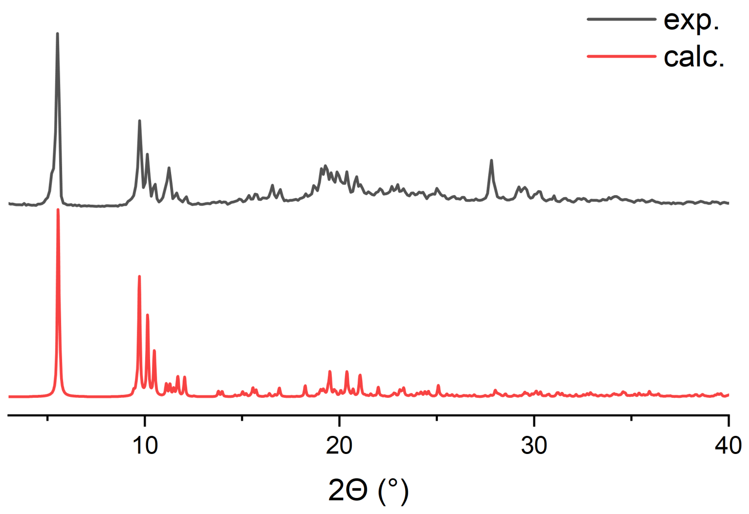

Figure S2. Calculated and experimental XRD patterns of MOF **1**.

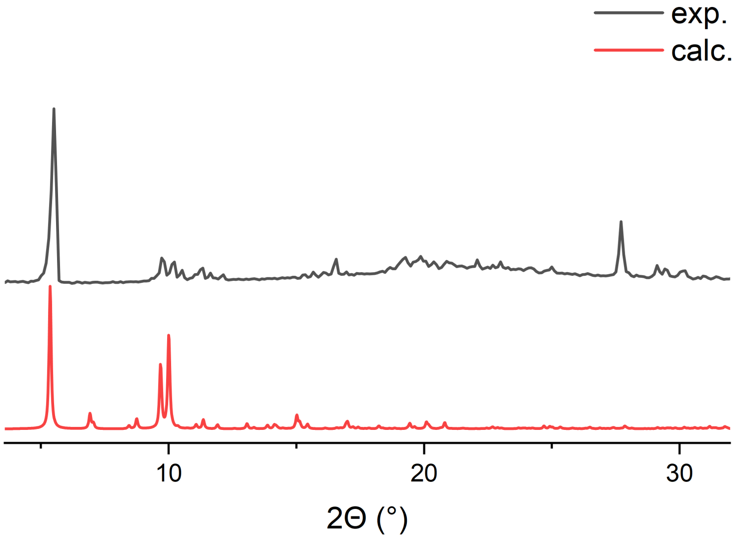

Figure S3. Calculated and experimental XRD patterns of MOF **2**.

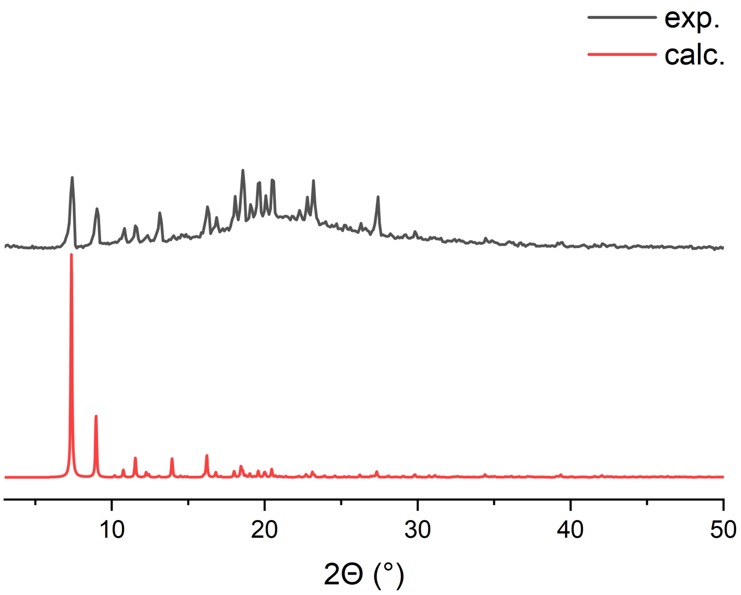

Figure S4. Calculated and experimental XRD patterns of MOF **3**.

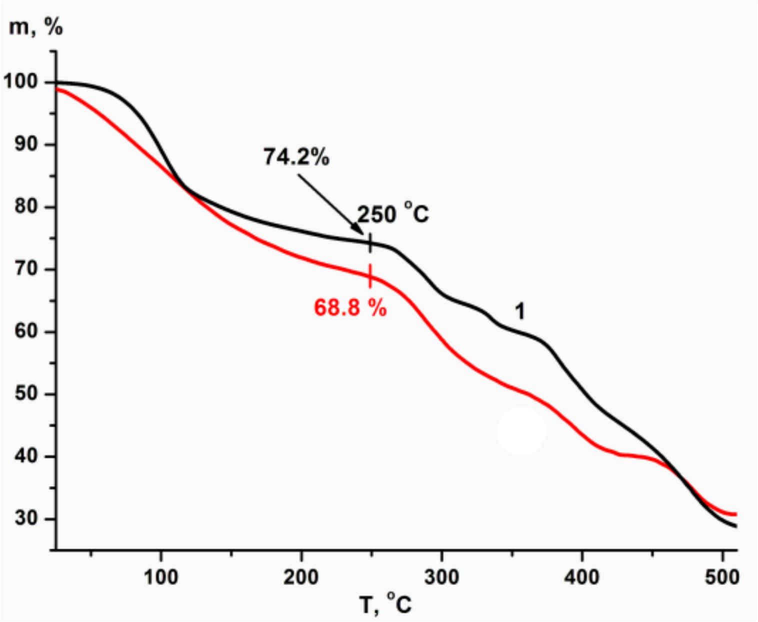

Figure S5. TGA curves of MOFs **1 and 3a**.

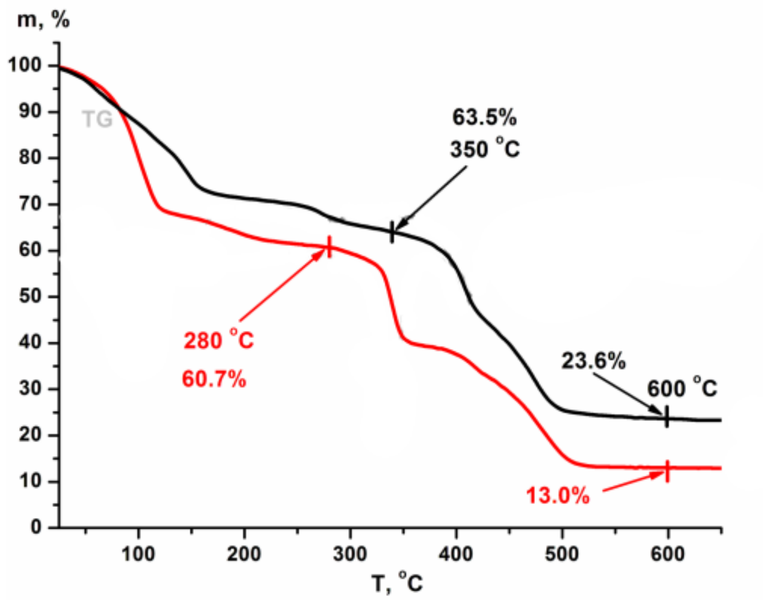

Figure S6. TGA curves of MOFs **1 and 3a**.

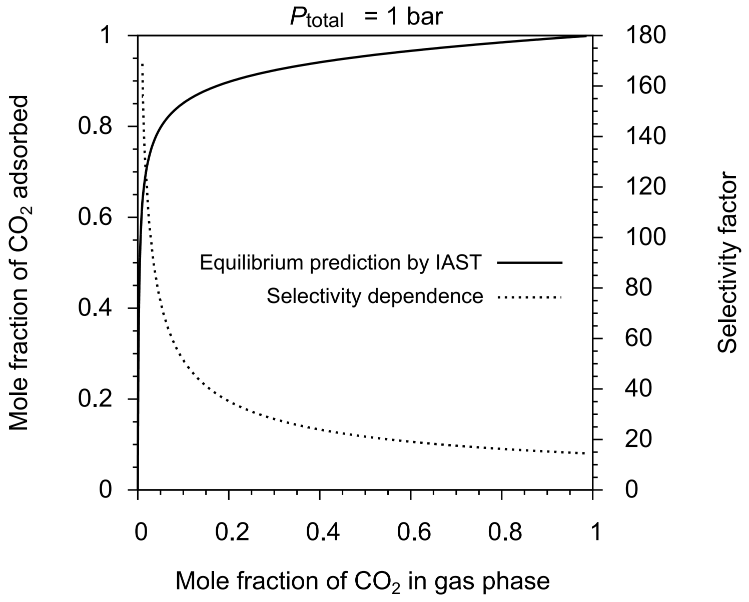

Figure S7. The adsorption equilibrium prediction by IAST for CO_2/N_2 binary mixture on MOF **1** (solid line) and the dependence of CO_2/N_2 adsorption selectivity (dotted line).

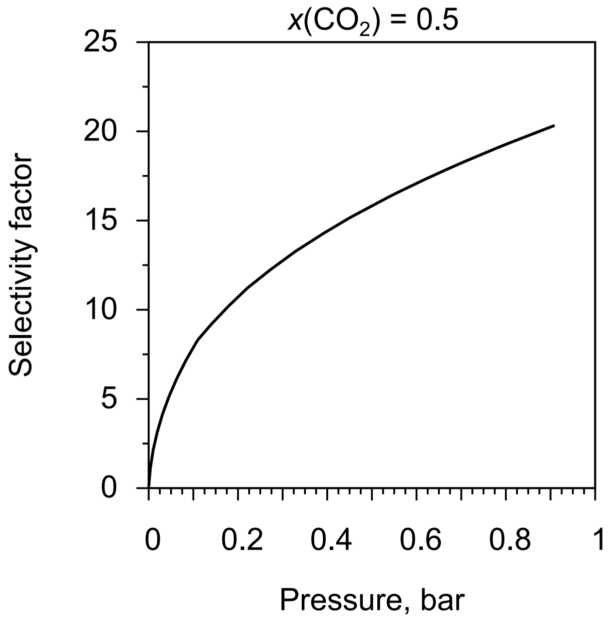

Figure S8. The dependence of CO_2/N_2 adsorption selectivity factor on the total pressure of equimolar gas mixture.

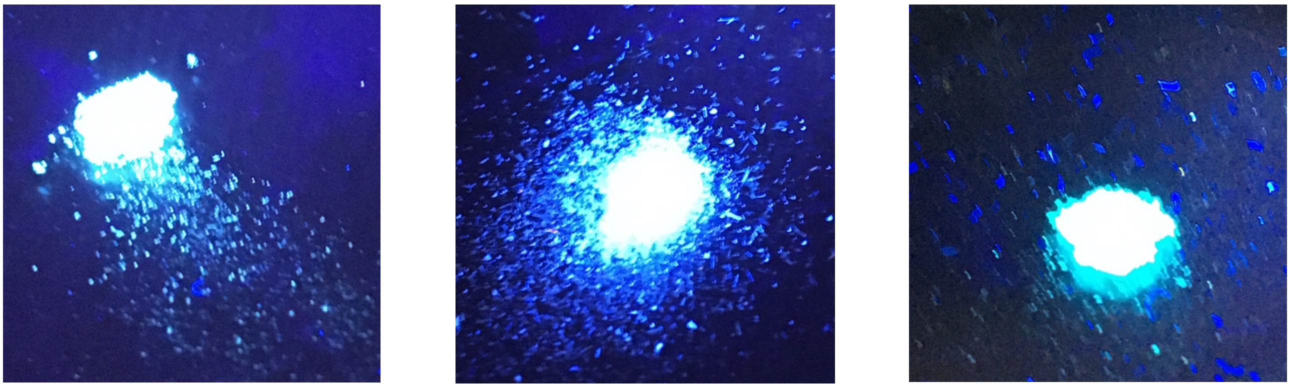

MOF **1** MOF **2** MOF **3**

Figure S9. Photographic images of the luminescence of MOFs 1-3 under 365 nm UV light.

Table S1. Carbon dioxide and nitrogen uptakes by MOF **1** at 1 bar and 273 K.

**Uptake**	**CO_2**	**N_2**
	**273 K**	**273 K**
**mL/g**	17.9	1.6
**mmol/g**	0.799	0.071

Table S2. Henry constants for gas adsorption by MOF **1** at 273 K.

**K_H**	**CO_2**	**N_2**
	**273 K**	**273 K**
**$mL·g^{-1}·torr^{-1}$**	0.094±0.007	$(2.489±0.011)·10^{-3}$
**$mmol·g^{-1}·bar^{-1}$**	3.15±0.23	0.08333±0.0004

Table S3. Adsorption isotherms models for IAST calculations.

**Compound**	**273 K**	
	**CO_2**	**N_2**
**1**	LF[a]<br>w = 28.43±0.19<br>$b = (7.90±0.11)·10^{-3}$<br>t = 1.235±0.007	L[b]<br>w = 8.5±0.4<br>$b = (3.13±0.18)·10^{-4}$

[a] Langmuir-Freundlich equation $n[\mathrm{mL/g}] = \frac{wbp[\mathrm{torr}]^{1/t}}{1+bp[\mathrm{torr}]^{1/t}}$;

[b] Langmuir equation $n[\mathrm{mL/g}] = \frac{wbp[\mathrm{torr}]}{1+bp[\mathrm{torr}]}$.

Table S4. Crystal data and structure refinement for the compounds.

Identification code	**1**·4.5DMF	**2**·8DMF	**3**·3DMF	**3a**·3DMF
Empirical formula	$C_{45.5}H_{56.5}N_{11.5}O_{17.5}Zn_3$	$C_{29}H_{18}N_6O_{12}Zn_3$	$C_{13}H_{10}N_6O_4Zn$	$C_{22}H_{31}N_9O_7Zn$
Formula weight	1240.67	1423.4	598.9	598.93
Temperature/K	150(2)	298(2)	298(2)	150(2)
Space group	*P*–1	*Pbca*	*C*2/*c*	$P2_1/c$
a/Å	9.7232(13)	20.1879(7)	23.893(3)	19.9831(15)
b/Å	18.204(3)	18.2491(6)	16.604(3)	16.6095(13)
c/Å	18.626(3)	32.9335(10)	17.746(3)	17.4898(13)
α/°	117.577(4)	90	90	90
β/°	102.500(5)	90	124.479(7)	102.142(2)
γ/°	92.264(5)	90	90	90
Volume/Å^3	2815.6(7)	12133.1(7)	5803.7(14)	5675.2(7)
Z	2	8	8	8
ρ_{calc}g/cm^3	1.463	1.558	1.371	1.402
μ/mm^{-1}	1.338	1.215	0.863	0.921
F(000)	1200.0	3360.0	1536.0	2496.0
Crystal size/mm^3	0.35 × 0.25 × 0.1	0.2 × 0.1 × 0.1	0.3 × 0.2 × 0.1	0.26 × 0.15 × 0.08
Radiation	MoK*α* (*λ* = 0.71073)	MoK*α* (*λ* = 0.71073)	MoK*α* (*λ* = 0.71073)	MoK*α* (*λ* = 0.71073)
2Θ range for data collection/°	4.348 to 51.638	3.192 to 48.81	3.208 to 48.806	2.084 to 48.812
Index ranges	-11 ≤ h ≤ 11<br>-22 ≤ k ≤ 22<br>-22 ≤ l ≤ 22	-23 ≤ h ≤ 22<br>-21 ≤ k ≤ 13<br>-38 ≤ l ≤ 38	-27 ≤ h ≤ 27<br>-19 ≤ k ≤ 19<br>-16 ≤ l ≤ 20	-23 ≤ h ≤ 23<br>-19 ≤ k ≤ 19<br>-20 ≤ l ≤ 20
Reflections collected	38194	70277	28348	57338
Independent reflections	10769 [R_{int} = 0.0238, R_{sigma} = 0.0235]	9982 [R_{int} = 0.0594, R_{sigma} = 0.0379]	4771 [R_{int} = 0.0577, R_{sigma} = 0.0413]	9328 [R_{int} = 0.0671, R_{sigma} = 0.0558]
Data/restraints/parameters	10769/51/601	9982/81/451	4771/24/239	9328/0/715
Goodness-of-fit on F^2	1.060	1.059	1.032	1.060
Final R indexes [I>=2σ (I)]	R_1 = 0.0577, wR_2 = 0.1695	R_1 = 0.0788, wR_2 = 0.2107	R_1 = 0.0668, wR_2 = 0.1997	R_1 = 0.0506, wR_2 = 0.1316
Final R indexes [all data]	R_1 = 0.0661, wR_2 = 0.1782	R_1 = 0.0889, wR_2 = 0.2172	R_1 = 0.0846, wR_2 = 0.2197	R_1 = 0.1102, wR_2 = 0.1681
Largest diff. peak/hole / e Å^{-3}	1.98/-1.15	0.80/-1.23	0.87/-0.64	0.70/-0.53
